# Ivermectin Bezoar: An Unusual Case Presentation

**DOI:** 10.7759/cureus.108424

**Published:** 2026-05-07

**Authors:** Samuel M Miller, Lucy Ruangvoravat, Felix Y Lui, Bishwajit Bhattacharya

**Affiliations:** 1 Surgery, Yale School of Medicine, New Haven, USA; 2 Surgery, West Haven Veterans Affairs (VA), West Haven, USA

**Keywords:** bezoar, gastrointestinal bleed, ivermectin, small bowel obstruction, surgery

## Abstract

This case report presents an older male who presented with small bowel obstruction after ingesting ivermectin horse paste that he had purchased online without a prescription. Unfortunately, ingestion of ivermectin by humans, including non-human formulations, has become more common after it was misrepresented as a treatment for COVID during the pandemic. This patient experienced a recurrent small bowel obstruction that resulted in life-threatening gastrointestinal bleeding and required multiple surgeries. As such, it should serve as a warning of the dangers involved in consuming medicines for off-label indications without direct supervision by a medical professional.

## Introduction

Ivermectin has become quite controversial since it was proposed as a treatment for and preventative measure against COVID-19 [1]. This case report describes a life-threatening complication that resulted from human ingestion of an ivermectin formulation intended for equine use.

Historically used to treat parasitic infections [2], ivermectin was reported to inhibit replication of the SARS-CoV-2 virus [3] and then touted by many as a treatment for COVID-19 [4]. As it gained popularity rapidly, the Federal Drug Administration (FDA) repeatedly advised against the use of ivermectin for the treatment of COVID-19 [5].

The FDA warned not only of the ineffectiveness of ivermectin for the treatment of COVID-19 but also of the significant risk of side effects. Oral ivermectin can cause nausea, vomiting, diarrhea, hypotension, headache, edema, and dizziness [6,7]. Multiple reports highlighting these side effects were published during this period of increased use [8,9]. Notably, many of the patients presenting with ivermectin toxicity in these reports had ingested veterinary formulations of ivermectin.

We discuss a rare case of a man who ingested an ivermectin paste formulated for horses and, several weeks later, presented with a small bowel obstruction. To the authors' knowledge, this is the first reported case of a bezoar forming from the congealed ivermectin paste. He experienced a life-threatening gastrointestinal bleed and required multiple surgeries during his admission. This case highlights the danger of taking medications without oversight from a medical professional as well as the impact that the mass media can have on medical decision-making.

## Case presentation

A 79-year-old male presented to the emergency department with two weeks of progressive nausea and abdominal pain. His last bowel movement was one week prior. He reported multiple bouts of emesis over the prior two days. He denied fevers, sick contacts, and weight loss. His only prior abdominal surgery was a laparoscopic cholecystectomy. His cancer screening was up to date. His last colonoscopy was three years prior, showing non-dysplastic tubular adenomas and sigmoid diverticulosis. His last stool prior to constipation was black. His vomitus was dark brown. Of note, he presented to an outside hospital several days prior with similar symptoms. CT scan of the abdomen/pelvis with oral contrast revealed constipation without evidence of intra-abdominal mass or obstruction, and he was discharged home. His medical history included coronary artery disease (CAD) on clopidogrel status post coronary artery bypass grafting (CABG) and transcarotid artery revascularization (TCAR), atrial fibrillation not on anticoagulation after Watchman procedure, and chronic kidney disease (CKD) not requiring dialysis.

Workup in the emergency department was notable for tachycardia to the 100s. He was normotensive and afebrile. His abdomen was distended with moderate diffuse tenderness. WBC was 14 with lactate at 1.6. Hemoglobin was 14.7 (normal: 13.5-17.5). Creatinine was elevated above his baseline, so a non-contrast CT of the abdomen/pelvis was obtained. This showed distended loops of small bowel with a transition point in the mid-pelvis. Oral contrast from his CT scan several days prior was present in the transverse colon. The patient was admitted to the surgical floor for bowel rest and intravenous hydration. Plavix was held.

**Figure 1:**
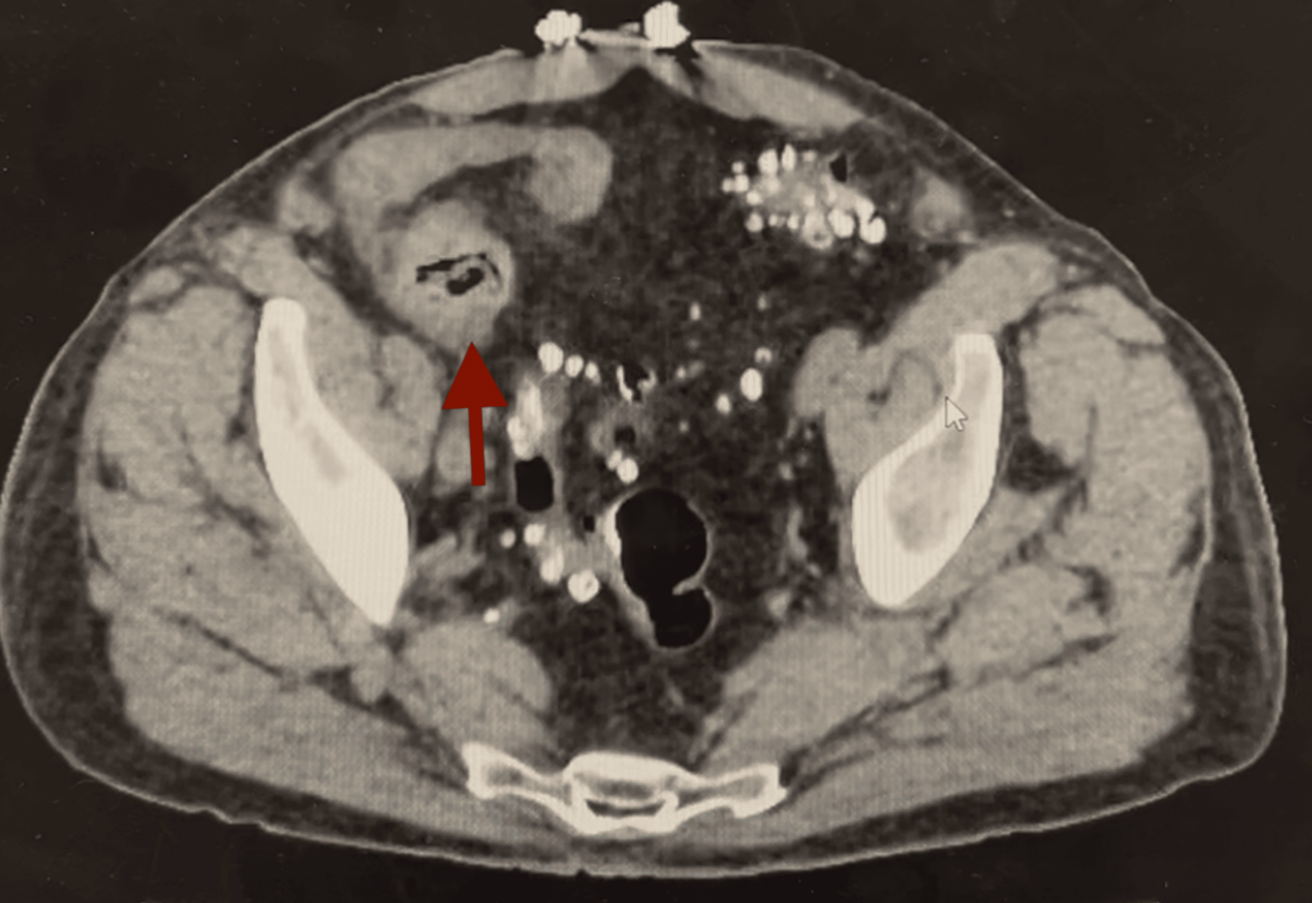
Preoperative CT protocolized for small bowel. Thickened segment of the ileum (arrow) and presumed site of obstruction.

Overnight on hospital day (HD) 2, the patient vomited, and a nasogastric tube was placed. He subsequently removed this tube and left the hospital against medical advice, only to re-present the next day (effectively HD3) with worsening abdominal pain. A small bowel follow-through series on HD4 showed progression of oral contrast to his colon. He had two liquid bowel movements that were not bloody. He tolerated food by mouth but was not passing flatus. The next morning, he had a large episode of feculent emesis. A nasogastric tube was placed with significant output. Over the next several days, he continued to have large-volume output from his nasogastric tube but reported consistent flatus. CT was repeated, this time protocolized to better evaluate the small bowel. This showed persistent small bowel obstruction with focal thickening in the distal ileum (Figure 1). We discussed his lack of improvement and the utility of surgery to investigate this concerning small bowel with the patient and his family. At this time, a family member voiced concern that the patient had ingested a tube of ivermectin horse paste just prior to the onset of his symptoms. Notably, the patient did not have any psychiatric history. Surgery was scheduled for HD8.

**Figure 2:**
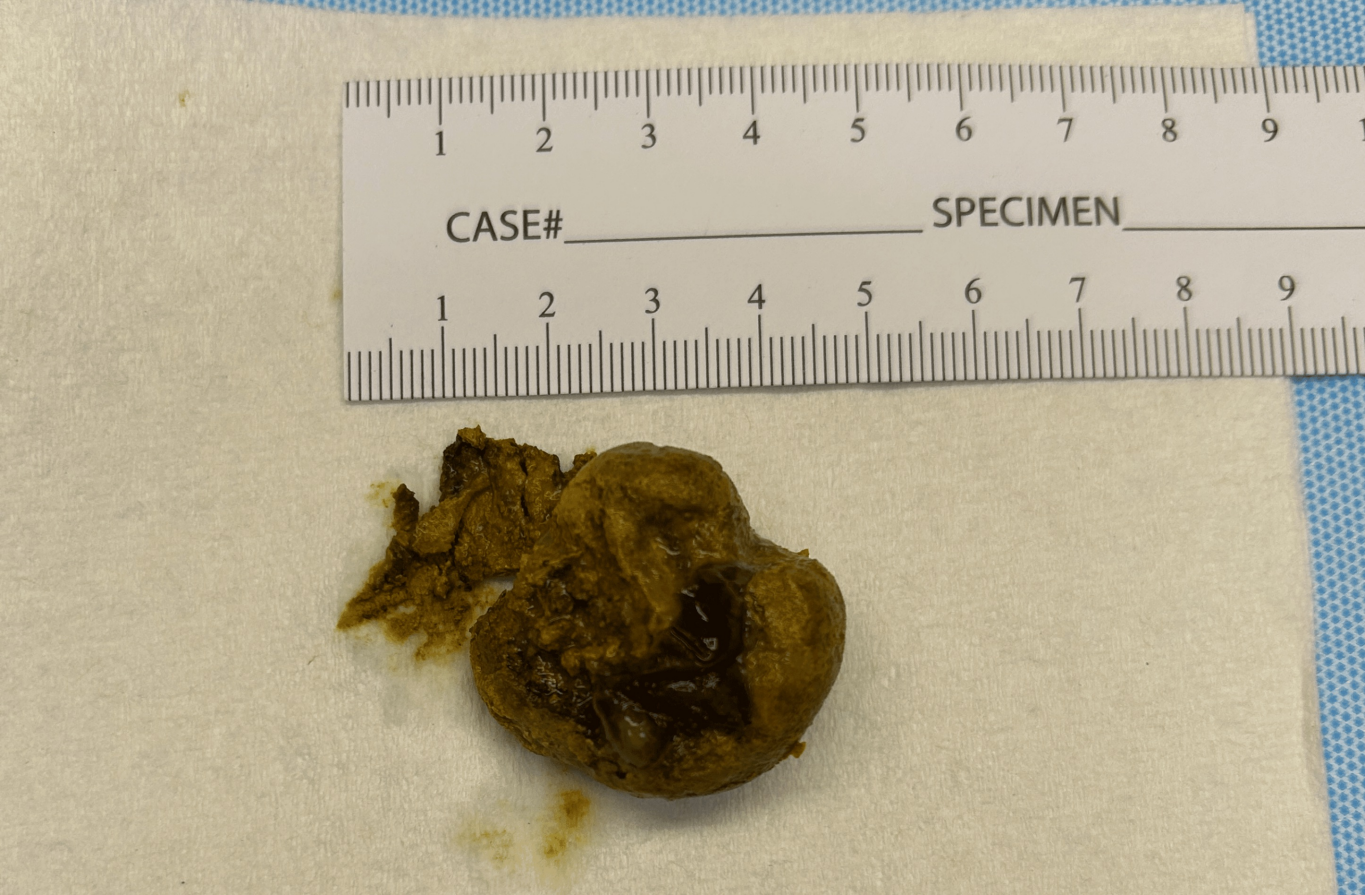
Bezoar removed from the small intestine. This bezoar measured approximately 4 cm in diameter. It was firm and mobile in the distal ileum.

The patient underwent an exploratory laparotomy. There was a small intraluminal mass in the distal ileum that was hard and mobile. The small bowel proximal to this mass was distended and injected, while the distal small bowel was decompressed and appeared healthy. The appendix, colon, stomach, and liver were unremarkable. The mass was removed through a 2 cm longitudinal enterotomy made just proximal to the mass. The mass was easily removed through the enterotomy (Figure 2). A small amount of bilious fluid was expressed, and hemostasis was confirmed. The enterotomy was closed transversely. A nasogastric tube was placed, and the abdomen was closed. The patient awoke from anesthesia uneventfully and returned to the surgical floor.

**Figure 3:**
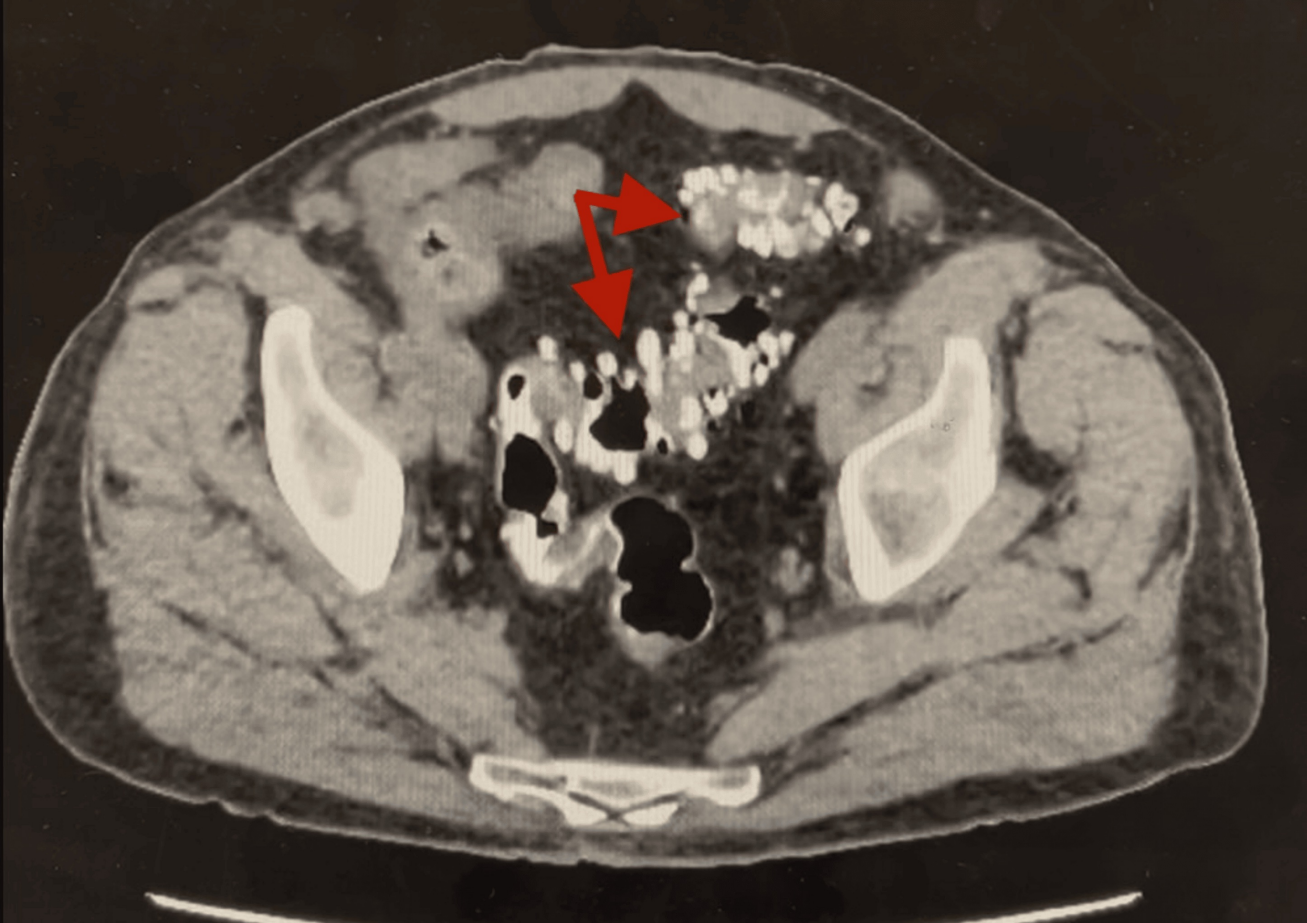
Preoperative CT showing extensive colonic diverticulosis. Arrows show colonic diverticuli with contrast uptake.

On postoperative day (POD) 0, a small amount of blood-tinged output was noted from the nasogastric tube. A daily dose of IV proton-pump inhibitor (PPI) was started. Twice daily dosing began on POD 1, given continued blood-tinged output. By POD 3, output was downtrending in volume and less bloody, prompting removal. Home medications were resumed, and he tolerated a clear liquid diet. He had a bowel movement the next morning with both dark and bright red blood. Hemoglobin dropped to 8.9 from 12.2. Repeat hemoglobin was 9.6 with stable hemodynamics. Later that evening, he had another bowel movement with dark and bright blood, this time with a drop in his systolic blood pressure to the 80s. Repeat CBC revealed hemoglobin of 7.1. He was transfused one unit of packed red blood cells (pRBCs) and made nothing per mouth (NPO). His blood pressure responded well, but his hemoglobin rose inadequately to only 7.9 the next morning. With continued bloody bowel movements, he was transfused a second and third unit of pRBCs. In addition to anastomotic bleeding, colonic bleeding was considered, given his extensive diverticulosis on previous CT imaging (Figure 3) and his lack of prior colonoscopy. He agreed to colonoscopy the next day.

**Figure 4:**
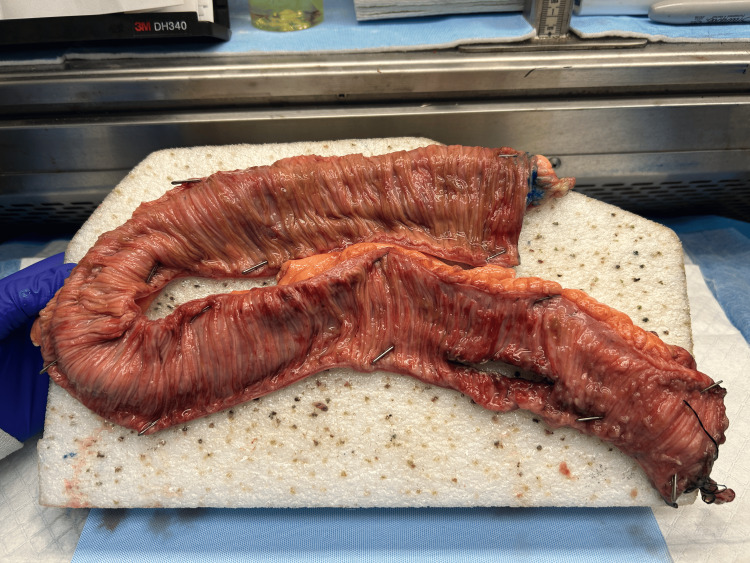
A 50-cm segment of resected small bowel. Stitch marks the distal margin on the bottom segment of the pictured small bowel. The enterotomy is seen at the screen right with mucosal pustules and hemorrhagic mucosa seen in the proximal small bowel.

**Figure 5:**
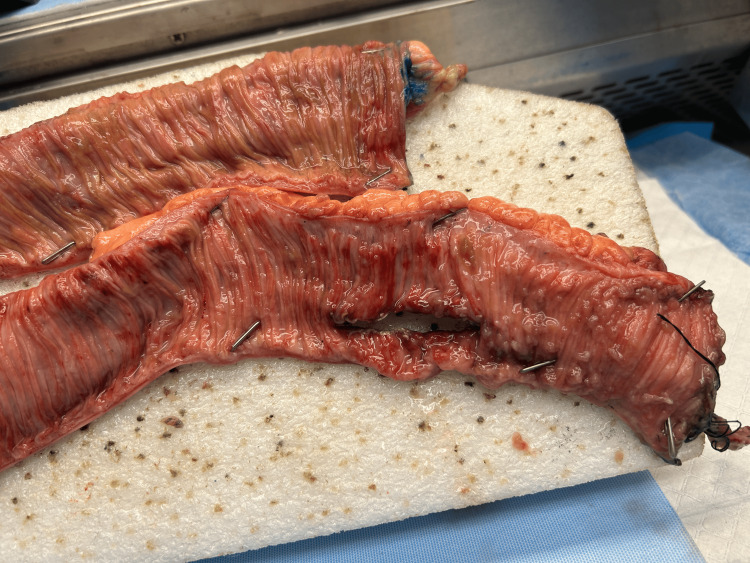
Close up of the resected small bowel. Stitch marks the distal margin on the bottom segment of the pictured small bowel. Again, the enterotomy is seen at the screen right with mucosal pustules and hemorrhagic mucosa seen in the proximal small bowel.

Colonoscopy was performed on POD 6. Blood was seen throughout the entirety of the colon as well as in the terminal ileum. After discussion with his family, we proceeded immediately to the operating room to identify the source of his bleeding. His previous laparotomy incision was extended to facilitate adequate exposure. The site of the previous enterotomy was identified and confirmed to be intact. The small bowel proximal to the enterotomy was distended and injected, while the distal small bowel appeared normal. The enterotomy was opened, and no obvious source of bleeding was identified. We were able to express a significant amount of clotted blood from the proximal small bowel. At this time, our GI colleagues joined us in the OR to perform small bowel enteroscopy. First, upper endoscopy was performed, which did not reveal a source of bleeding in the stomach or proximal duodenum. We proceeded to manipulate the small bowel in the operative field to facilitate the progression of the endoscope through the small bowel. Blood clots were identified in the distal jejunum. Once these clots were cleared, we were able to visualize multiple ulcers that appeared to be ischemic. These ulcers were present throughout the remaining distal jejunum and the entirety of the ileum until the endoscope advanced through the previous enterotomy. The ulceration was denser in the area just proximal to the enterotomy. The scope was then withdrawn, and a stapled resection of the small bowel containing the enterotomy and the densest ulceration was resected. A stapled anastomosis was performed. In total, just over 50 cm of small bowel was resected (Figure 4). Pathology noted mucosal pustules and hemorrhagic mucosa throughout the specimen, with normal mucosa at the proximal end and the small bowel distal to the enterotomy (Figure 5).

The patient recovered and was extubated and weaned from pressors by POD 3. He did not require blood transfusions postoperatively and slowly recovered bowel function. He was discharged to a skilled nursing facility from the hospital. The bezoar matter was sent to the Animal Health Diagnostic Center at the College of Veterinary Medicine at Cornell University, where it was confirmed to contain ivermectin material.

## Discussion

This patient presented with an intermittent small bowel obstruction and was found to have a bezoar in the distal ileum composed of ivermectin matter. The bezoar was formed by congealed ivermectin horse paste, which he purchased online and ingested prior to presentation. To these authors’ knowledge, this is the first reported case of ivermectin ingestion requiring operative intervention.

The obstruction caused by this ivermectin bezoar led to prolonged exposure of the small bowel mucosa to the ivermectin paste, which, in these authors' opinion, caused the diffuse ulceration and gastrointestinal bleeding. It is important to acknowledge other possible etiologies of the observed pathology, including pressure injury from the bezoar and localized ischemia from luminal distention. In addition, the degree of observed mucosal hemorrhage may have been due to the persistent effects of clopidogrel.

After his first surgery, a colonoscopy was crucial in identifying the source of this bleed as the small bowel, as opposed to the colon. During his second surgery, on-table small bowel enteroscopy allowed for the identification of ulcerated mucosa as the source of bleeding. This is not a common procedure, but it is very helpful in visualizing the small bowel not accessible with standard upper endoscopy [10]. Pathologic analysis revealed areas of focal ulceration consistent with acute ischemic injury. We hypothesize that the ingested paste congealed to form a bezoar, which caused the mechanical obstruction. The remaining ivermectin paste was therefore unable to move forward and remained in the proximal small intestine for several weeks, resulting in toxic exposure and mucosal injury.

Ivermectin toxicity, though rare when taken in accordance with pharmaceutical guidelines, is well documented. Gastrointestinal symptoms are most common and typically begin within two hours of a single, large, first-time dose [9]. Notably, many of the patients presenting with ivermectin toxicity in these reports ingested veterinary formulations of ivermectin. The FDA specifically advises against taking ivermectin meant for animals, explaining “due to the lack of testing in humans, the safety of these products in humans is not known” [7]. Horse formulations of ivermectin are known to include significantly higher doses of the drug, given that horses are much larger than humans [11].

Our patient was unaware of these potential side effects when he purchased the ivermectin horse paste. He could not remember if he had been feeling unwell prior to his ingestion, but recounted watching videos online about the benefits of ivermectin. His primary care physician refused to prescribe ivermectin, and so he resorted to a more easily accessible veterinary formulation. Unfortunately, the non-prescription use of ivermectin has been seen more frequently in older people and those with the highest degree of social vulnerability [12]. This was the case for our patient, and he described his embarrassment as influencing his decision to wait two weeks before seeking medical care.

## Conclusions

This case highlights the potential for life-threatening complications related to ivermectin ingestion. Our patient presented after ingesting ivermectin horse paste and ultimately experienced mechanical obstruction due to a bezoar, gastrointestinal bleeding, prolonged intubation, and multiple surgeries. To the authors’ knowledge, this is the first case of ivermectin ingestion causing a small bowel obstruction that required operative intervention. It is important that patients understand the potential risks involved in taking this medicine, especially without medical supervision.
